# 2118 Solid-state MRI as a nonradiative alternative to computed tomography for craniofacial imaging

**DOI:** 10.1017/cts.2018.123

**Published:** 2018-11-21

**Authors:** Rosaline Zhang, Hyunyeol Lee, Xia Zhao, Hee K. Song, Felix W. Wehrli, Scott P. Bartlett

**Affiliations:** 1 University of Pennsylvania School of Medicine, Philadelphia, PA, USA; 2 Division of Plastic Surgery, Children’s Hospital of Philadelphia, University of Pennsylvania, Philadelphia, PA, USA; 3 Department of Radiology, University of Pennsylvania, Philadelphia, PA, USA

## Abstract

OBJECTIVES/SPECIFIC AIMS: Computed tomography (CT) enables 3-dimensional (3D) visualization of cortical bone structures with high spatial resolution, and thus has been the gold-standard method for evaluation and diagnosis of craniofacial skeletal pathologies. However, ionizing radiation and, in particular, repeated scanning for presurgery and postsurgery assessments, is of concern when applied to infants and young children. Recent advances in solid-state MRI allow the capture of the short-T2 signals in cortical bone while suppressing the signal from soft-tissue protons having T2 relaxation time 1–2 orders of magnitude longer (50–100 ms). One approach, a dual-radiofrequency (RF) pulse and ultrashort echo time (UTE) imaging based method, exploits different sensitivities of bone and soft tissue to different RF pulse widths and TEs. This study aims to demonstrate the feasibility of producing 3D renderings of the human skull and visualization of cranial sutures using the bone-selective MRI technique in comparison to CT. METHODS/STUDY POPULATION: Imaging technique: Two RF pulses differing in duration and amplitude are alternately applied in successive repetition time (TR) along the pulse train. Within each TR, 2 echoes are acquired. Acquisition of the first echo starts at the ramp-up of the encoding gradient (TE1), allowing for capture of signals with very short lifetimes (bone), while that of the second starts after a longer delay (TE2). In total, 4 echoes are obtained: ECHO11 (RF1TE1), ECHO12 (RF1TE2), ECHO21 (RF2TE1), and ECHO22 (RF2TE2). During reconstruction, ECHO11 is combined with ECHO21 and ECHO12 is combined with ECHO22, resulting in 2 images. The subtraction of these 2 images yields an enhanced bone contrast. Data acquisition/processing: The pulse sequence described above was applied for MR imaging of a human cadaveric skull and 2 adult human subjects in vivo, at 3T field strength (Siemens Prisma, Erlangen, Germany). Imaging parameters: TR/TE1/TE2=7/0.06/2.46 ms, RF1/RF2 durations=40/520 μs, flip angle=12°, matrix size=2563, field of view=2803 mm^3^, voxel size=1.1 mm isotropic, number of radial spokes=25,000, and scan time=6 minutes. Segmentation of bone voxels was performed using ITK-SNAP in a semi-automatic fashion, leading to 3D renderings of the skull. For comparison, a CT scan was also performed in the human cadaveric skull with 1 mm isotropic resolution. Validation: The biometric accuracy was assessed by measuring eight anatomic distances: (1) Maximum craniocaudal aperture of the right orbit. (2) Maximum craniocaudal aperture of the left orbit. (3) Maximum height of the mandible from chin point in the midline. (4) Maximum cranial length (5) Maximum cranial width. (6) Maximum height of piriform aperture. (7) Distance between lateral most aspect of mandibular condyles. (8) Distance between lateral most aspect of posterior hard palate in both CT- and MRI-based 3D renderings of the human cadaveric skull using Mimics software (Materialise^®^, Ghent, Belgium). These distances were compared with those directly measured on the cadaveric skull. RESULTS/ANTICIPATED RESULTS: Compares CT with the proposed MRI method on cadaveric human skull images, along with corresponding 3D renderings. Compared with CT, the 3D rendered images maintain most features over the entire head (e.g., zygomatic arch), except for appearance of some artifacts in the mandibular region. In vivo head images in 2 adult subjects: axial magnitude images and 3D rendering. In the axial images, bone voxels as well as the inner table of the cranium are clearly visualized, and cranial and spinal bone structures are well depicted in the 3D renderings. Some voxels were erroneously included or excluded in the renderings. The mean difference in measurements of the 8 anatomic distances was 6, 4, and 2 mm when comparing MRI Versus CT, MRI Versus in situ, and CT Versus in situ, respectively. DISCUSSION/SIGNIFICANCE OF IMPACT: Bone proton magnetization exhibits a substantial level of signal decay during the relatively long duration of RF2 due to its very short T2 relaxation time. In contrast, soft-tissue retains nearly the same level of signal intensities over all echoes. Thus, subtraction of ECHO22 from ECHO11, when compared with the difference between ECHO11 and ECHO12, enhances bone contrast from soft tissue. The proposed, dual-RF dual-echo 3D UTE imaging technique produces isotropic high-resolution bone-specified images in the whole head within a clinically feasible imaging time (6 min), leading to clear visualization of craniofacial skeletal structures. These are key components necessary for translation to the clinical setting. Optimization of postprocessing for more realistic 3D renderings and thus accurate anatomic measurements is currently being implemented. The proposed method’s potential as a nonradiative alternative to CT will then be thoroughly evaluated in pediatric patients.

